# Comparative analysis of molecular signatures reveals a hybrid approach in breast cancer: Combining the Nottingham Prognostic Index with gene expressions into a hybrid signature

**DOI:** 10.1371/journal.pone.0261035

**Published:** 2022-02-10

**Authors:** Dimitrij Tschodu, Bernhard Ulm, Klaus Bendrat, Jürgen Lippoldt, Pablo Gottheil, Josef A. Käs, Axel Niendorf

**Affiliations:** 1 Peter Debye Institute, Leipzig University, Leipzig, Germany; 2 Independent Statistical Consulting Bernhard Ulm, Munich, Germany; 3 MVZ Prof. Dr. med. A. Niendorf Pathologie Hamburg-West GmbH, Institute for Histology, Cytology and Molecular Diagnostics, Hamburg, Germany; Universita degli Studi di Torino, ITALY

## Abstract

The diagnosis of breast cancer—including determination of prognosis and prediction—has been traditionally based on clinical and pathological characteristics such as tumor size, nodal status, and tumor grade. The decision-making process has been expanded by the recent introduction of molecular signatures. These signatures, however, have not reached the highest levels of evidence thus far. Yet they have been brought to clinical practice based on statistical significance in prospective as well as retrospective studies. Intriguingly, it has also been reported that most random sets of genes are significantly associated with disease outcome. These facts raise two highly relevant questions: What information gain do these signatures procure? How can one find a signature that is substantially better than a random set of genes? Our study addresses these questions. To address the latter question, we present a hybrid signature that joins the traditional approach with the molecular one by combining the Nottingham Prognostic Index with gene expressions in a data-driven fashion. To address the issue of information gain, we perform careful statistical analysis and comparison of the hybrid signature, gene expression lists of two commercially available tests as well as signatures selected at random, and introduce the Signature Skill Score—a simple measure to assess improvement on random signatures. Despite being based on *in silico* data, our research is designed to be useful for the decision-making process of oncologists and strongly supports association of random signatures with outcome. Although our study shows that none of these signatures can be considered as the main candidate for providing prognostic information, it also demonstrates that both the hybrid signature and the gene expression list of the OncotypeDx signature identify patients who may not require adjuvant chemotherapy. More importantly, we show that combining signatures substantially improves the identification of patients who do not need adjuvant chemotherapy.

## Introduction

Important questions arise when considering a breast cancer (BC) tumor: What is the survival probability? How can one assess the usefulness of additional adjuvant measures? BC remains the most frequent malignancy in women worldwide [1], and to answer these questions clinicians can take two major approaches.

Traditionally, prognostication has hinged on clinical and pathological characteristics. The most prominent example of this approach represents the Nottingham Prognostic Index (NPI) [2–4], the current version of which includes lymph node involvement, tumor stage and grade, assembling these in a simple prognostic index formula. The resulting NPI scores are then used to stratify patients into risk groups.

On the other hand, technological advances have brought remarkable achievements in cancer genetics such as rapid sequencing of the entire human genome and cataloging vast amounts of mutations, gene expressions, protein levels and overall genetic profiles: the Cancer Genome Atlas being the most promising project in cancer research [5]. This project has led thousands of researchers to reconsider cancer on the molecular level, envisioning “precision oncology” as the logical next step in cancer therapy. Precision oncology is based on the idea that so-called biomarkers or molecular signatures can predict disease phenotype, patient outcome, and therapy response—independently of tumor histology. However, currently less than 25% of patients with the most frequent cancers benefit from precision oncology [6].

Over 150,000 papers documenting new molecular signatures have been produced in medicine, but only fewer than 100 have been clinically validated [7]. The 2014 European Society of Medical Oncology clinical practice guidelines for breast, lung, and colon cancer recognized less than 20 biomarkers with sufficient clinical evidence [8]. Especially in BC, evaluating gene signatures presents a serious problem of “overtrust” [9], in which the output of a prediction algorithm is treated as indubitable rather than used as a source of aid.

**Fig 1:**
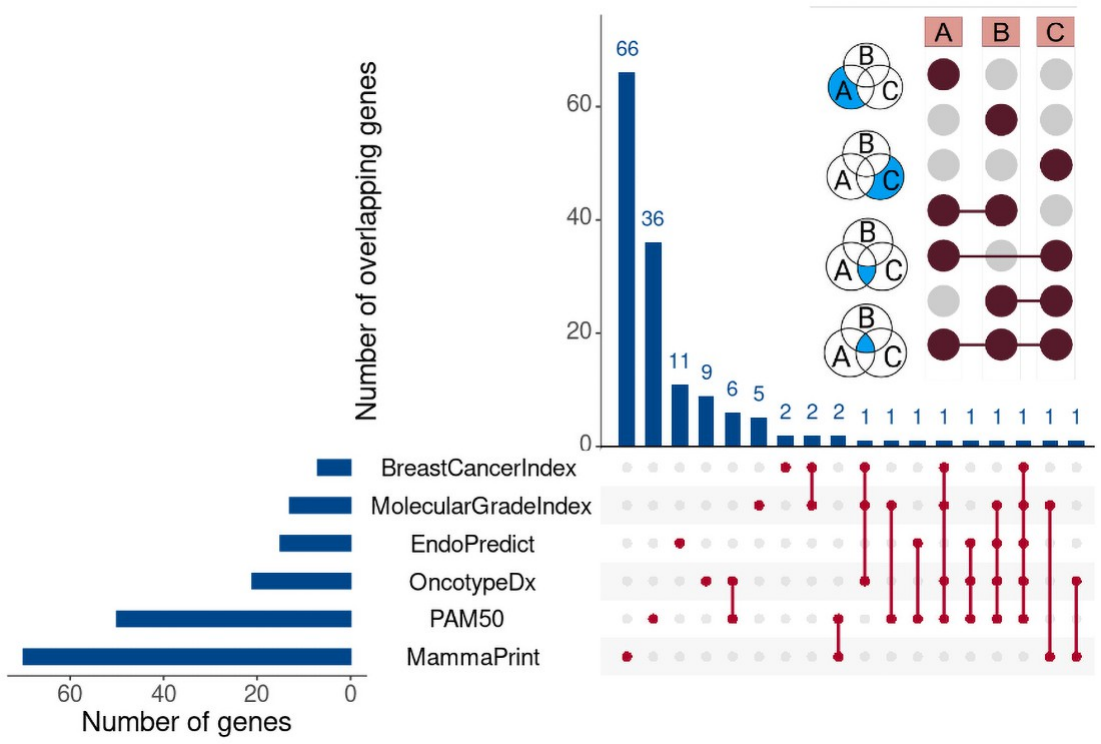
UpSet diagram for overlapping genes of six commercially available signatures [15]. Horizontal bars represent the number of genes in signature. Vertical bars indicate the number of overlapping genes. The inset graphic illustrates the interpretation for 3 sets: Connected dots indicate exclusive overlapping and an unconnected dot represents exclusive non-overlapping. For example, a single unconnected dot for MammaPrint means that 66 genes are contained only in this signature and are not shared in any combination with other signatures. Two connected dots for PAM50 and OncotypeDx denote that 6 genes are exclusively shared between these signatures. As can be seen, the overlapping is almost non-existing. In this study, we used the gene lists of EndoPredict and OncotypeDx for comparison, since they are the most prominent signatures.

By now, there are at least six commercially available gene signatures for BC: OncotypeDx, EndoPredict, MammaPrint, Genomic Grade Index, PAM50, and Breast Cancer Index. Yet, a low level of agreement (*κ* = 0.40, 95% CI: 0.30x2013; 0.49) between MammaPrint and OncotypeDx was found in the recent OPTIMA study [10], which impairs their use for routine clinical practice. Moreover, overlap among the six signatures is almost non-existent: Fig 1 demonstrates that not a single gene is shared by all of the six signatures. Showing moderate concordance values [11, 12], none if these signatures seems to have reached the highest level of evidence [13]. Intriguingly, Venet *et al.* reported that any set of 100 or more randomly selected genes has a probability of 0.9 to be significantly associated with patient outcome [14].

Thus, a natural question to ask is: Can one combine the clinical and pathological characteristics with the molecular characterization? If so, can we find an algorithm for selecting genes that significantly improves prediction compared with random sets as negative controls? For this purpose, we treated the NPI score as a gene expression and used it along with other gene expressions for training an algorithm that selected the NPI among other genes.

We call this emerged feature set *Hybrid signature*. Next, we compared the Hybrid signature with the two most prominent gene signatures, OncotypeDx and EndoPredict, and randomly generated signatures.

It is believed that current clinical and pathological assessment gives rise to overtreatment that is detrimental to the patient [16]. The TAILORx and the MINDACT studies have shown that OncotypeDx and MammaPrint provide guidance for adjuvant chemotherapy, respectively [17–19]. We selected patients *in silico* that were not subjected to chemotherapy.

Multigene expression assays suffer from low interchangeability and comparability [20]. We approximated OncotypeDx and EndoPredict by using corresponding gene lists to compare the inherent power of genes contained in each signature. Specifically, we took expressions of corresponding genes as input for our statistical models and did not use the algorithms applied by OncotypeDx or EndoPredict. We refer to these approximated signatures as OncotypeDxGL and EndoPredictGL (GL for Gene List).

## Materials and methods

The R code to reproduce the numerical results provided in the *Results* section can be found on GitHub located at the address: https://github.com/DiTscho/HybridSignature.

### Data description

We used gene expression data with annotated clinical data from the METABRIC (MB) cohort. It contains clinical and pathological information on patients’ long-term survival (up to 30 years) as well as expression measurements of 24,000 genes. Hosted by the European Bioinformatics Institute, all data can be downloaded from the EuropeanGenome-Phenome Archive at http://www.ebi.ac.uk/ega under accession number EGAS00000000083. The gene expression values were measured on the Illumina HT-12 v3 platform, already preprocessed and *log*_2_-normalized, as described in [21]. The function *avereps* in the R package *limma* was used to summarize genes with multiple probes [22]. The R package *illuminaHumanv3.db* was used to annotate genes [23]. Genes without annotations were removed. From the initial 2136 samples we selected 1262 samples of estrogen-receptor positive (ER+), Her2-receptor negative (Her2-) patients who did not receive cytotoxic chemotherapy, and who either died due to the disease or are still alive. These data were then randomly divided in training set and test set 1 with 883 (70%) and 379 (30%) samples, respectively. S1 Table summarizes the descriptive statistics for these datasets.

The second dataset was used as the external test set. It is publicly available in Gene Expression Omnibus [24] under the access number GSE96058. The expression matrix contains preprocessed *log*_2_-normalized expression values of a prospective population-based series of 3,273 BC patients with a median follow-up of 52 months (Sweden Cancerome Analysis Network—Breast [SCAN-B], ClinicalTrials.gov identifier: NCT02306096), as described in [25]. No further filtering was conducted. From the initial 3,273 samples we selected 1381 samples of estrogen-receptor positive (ER+), Her2-receptor negative (Her2-) patients with overall survival who did not receive cytotoxic chemotherapy. The overall survival was used, however, unlike in the METABRIC dataset no information about the cause of death is provided in the GSE96058 dataset. Consequently, we selected samples whose age was under 80 years to maximize the number of patients who died due to the disease. These data were then randomly downsampled to the second test set, which we denote as test set 2. We performed downsampling, i.e. a subset of patients was randomly sampled with the same event-to-patients-at-risk ratio as in the training set and test set 1. We repeated this procedure 1000 times and the corresponding results can be seen in S2 Appendix. S2 Table summarizes the descriptive statistics for these datasets.

We used ComBat in the R package *sva* [26] to adjust data for batch effects prior to analysis.

### Study design

**Fig 2:**
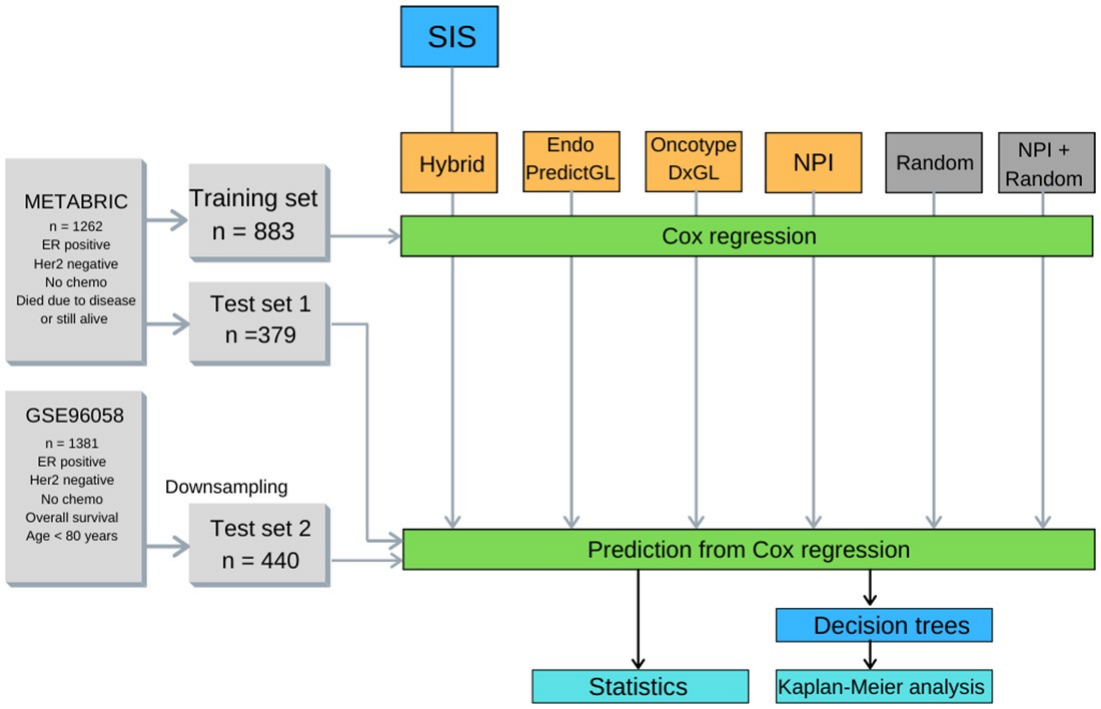
Study design. In the METABRIC cohort, we select 1262 estrogen-receptor positive, Her2-receptor negative patients who did not receive chemotherapy and who either died due to the disease or are still alive. This cohort is divided into training set and test set 1 with 883 (70%) and 379 (30%) patients, respectively. The training set is used to train the Sure Independence Screening algorithm (SIS). SIS selects 15 most important genes—including the Nottingham Prognostic Index (NPI)—that we call Hybrid signature. We select gene lists used by EndoPredict and OncotypeDx (not the corresponding recurrence or risk scores), and refer to them as EndoPredictGL and OncotypeDxGL. We also select the NPI scores alone. For comparison, 15 genes are selected at random (Random signature). To compare the Hybrid signature with a direct negative control, one gene in the Random signature is substituted with the NPI (NPI+Random). Using the training set, all six signatures are then subjected to Cox regressions. In the GSE96058 cohort, we select 1381 estrogen-receptor positive, Her2-receptor negative patients with overall survival who did not receive chemotherapy and who was younger than 80 years. Subsequently, we perform downsampling to ensure the same event-to-patients-at-risk ratio as in the training set and test set 1. We denote this downsampled set as test set 2. Predictions are made on the test set 1 and test set 2. Finally, we statistically compare the predictions and use Decision Trees to assess the survival analysis.

Our research question is whether we can construct a signature whose prediction performance is substantially better than the performance of competing signatures. With this question in mind, we use the Sure Independence Screening (SIS) [27]: an algorithm that finds most important genes with high probability. SIS is employed to rank and select the 15 most important genes based on gene expressions and survival times in the MB training set. The resulting gene set is then selected in the test set 1 as well as in the test set 2. Further, we select genes used by the multigene assays EndoPredict and OncotypeDx, and use the NPI scores provided in the MB data. We compare and challenge the results not with the null model of absence of any predictors, but with the results of 15 randomly sampled genes (“Random” in Fig 2). In addition, we construct a “hybrid random” signature by substituting one gene in the random sample with the NPI. This signature serves as a comparable negative control to the Hybrid signature (“NPI + Random” in Fig 2).

Finally, corresponding Cox regressions are estimated. The results are followed by the statistical analysis, along with the stratification of patient outcomes that was assessed by Decision Trees and survival analysis. The process is shown in Fig 2 and further described in the remainder of this section.

### Feature selection using SIS

A feature selection method tries to answer the question: Which features are the most important to predict the response, i.e. the patient outcome? “Features” are also called predictor variables or covariates, and “response” is also called the dependent variable. For example, one might try to find the genes (covariates) whose expressions are the most important to predict the survival outcome or survival time (responses).

Many methods such as Dantzig Selector, Adaptive Lasso, SCAD, or Bridge selection have been developed to address this question [27]. However, these methods may be unstable or may not satisfy the condition of finding the most important covariates [28, 29]. Further, there are two relevant problems in the context of gene expressions: Some unimportant genes may be correlated with important genes; and some genes may be not individually but collectively correlated with the outcome. To address these problems we used the (iterative) Sure Independence Screening [27]. In the context of SIS, the most important variables “survive”, i.e. they are selected after screening with the probability tending to one as the sample size increases (Theorem 3 in [27]).

We performed feature selection using the package *SIS* in the R software [30, 31]. Thereby, we performed the aggressive version of variable screening using “cox” as response type, “lasso” as penalty, and “bic” as tuning option for the regularization parameter.

### Gene selection

Please note, we selected gene lists used by EndoPredict and OncotypeDx and did not compute respective risk and recurrence scores, since they are platform-dependent. We refer to these gene lists as EndoPredictGL and OncotypeDxGL.

**EndoPredictGL**. The genes used by the EndoPredict assay [32] were selected. This gene signature predicts the high- or low-risk of recurrence ER-positive, HER2-negative in breast cancer treated with adjuvant endocrine therapy. The corresponding genes are listed in Table 1b.

**Table 1:** Cox proportional analysis of competing signatures in the METABRIC training set (n = 883). The 1st column lists gene names used in each signature. NPI: Nottingham Prognostic Index, HR: Hazard Ratio, CI: Confidence Interval.

	HR	95% CI	p-value
**(a)** Hybrid			
CDC20	1.46	1.05, 2.05	0.03
CLSPN	0.76	0.61, 0.95	0.02
SYTL4	0.90	0.76, 1.07	0.24
NUSAP1	1.48	0.96, 2.29	0.08
MELK	1.34	0.94, 1.91	0.10
CEP55	0.53	0.34, 0.83	0.01
CKAP2L	1.26	0.79, 2.02	0.33
PTTG1	1.26	0.86, 1.85	0.24
IRF3	1.66	1.14, 2.41	0.01
PREX1	0.62	0.51, 0.75	<0.001
OR5M11	2.65	1.04, 6.76	0.04
CCNB2	0.60	0.36, 1.00	0.05
NLRP1	0.66	0.48, 0.89	0.01
BUB1	1.27	0.81, 2.01	0.30
NPI	1.54	1.32, 1.80	<0.001
**(b)** EndoPredictGL			
ESR1	0.88	0.76, 1.03	0.10
ERBB2	0.71	0.52, 0.97	0.03
BIRC5	1.61	1.25, 2.08	<0.001
RBBP8	0.77	0.61, 0.96	0.02
UBE2C	1.22	0.93, 1.61	0.16
IL6ST	1.06	0.85, 1.33	0.60
AZGP1	1.00	0.88, 1.13	1.00
DHCR7	0.83	0.67, 1.02	0.07
MGP	0.97	0.88, 1.07	0.59
STC2	0.97	0.89, 1.06	0.51
CALM2	1.10	0.68, 1.77	0.71
PPIA	0.85	0.54, 1.35	0.49
OAZ1	1.43	0.87, 2.35	0.15
RPL37A	0.86	0.58, 1.28	0.47
PAEP	1.03	0.87, 1.21	0.76
**(c)** OncotypeDxGL			
MKI67	1.15	0.89, 1.48	0.30
AURKA	0.95	0.68, 1.31	0.74
BIRC5	1.45	1.10, 1.90	0.01
CCNB1	1.22	0.91, 1.62	0.18
MYBL2	0.90	0.78, 1.03	0.12
ERBB2	0.62	0.41, 0.92	0.02
GRB7	1.29	0.81, 2.06	0.28
ESR1	0.89	0.76, 1.04	0.14
PGR	0.83	0.76, 0.92	<0.001
BCL2	1.04	0.82, 1.32	0.73
SCUBE2	1.09	1.00, 1.20	0.06
CTSV	1.17	0.91, 1.51	0.23
MMP11	1.02	0.90, 1.16	0.71
BAG1	0.76	0.57, 1.02	0.07
CD68	0.90	0.69, 1.16	0.40
GSTM1	0.96	0.91, 1.02	0.18
ACTB	1.76	1.17, 2.66	0.01
GUSB	0.83	0.60, 1.14	0.24
GAPDH	1.24	0.86, 1.76	0.25
RPLP0	0.84	0.60, 1.18	0.32
TFRC	0.84	0.67, 1.05	0.13
**(d)** Random			
CADPS2	1.07	0.86, 1.34	0.54
PLAU	1.38	1.15, 1.65	<0.001
ACTR3C	0.92	0.74, 1.16	0.49
PHKG1	0.75	0.52, 1.08	0.13
RSPRY1	1.47	1.00, 2.17	0.05
P2RX6P	0.90	0.77, 1.05	0.18
TIGD6	1.10	0.71, 1.70	0.66
SPON1	0.72	0.61, 0.85	<0.001
PTPN14	1.32	0.96, 1.82	0.09
PDZK1	0.91	0.85, 0.99	0.02
BTBD10	1.64	1.02, 2.62	0.04
SLC9B2	0.74	0.57, 0.95	0.02
TRIM71	0.84	0.45, 1.59	0.60
ADCY6	0.82	0.59, 1.14	0.24
ALOX15B	0.96	0.85, 1.07	0.45
**(e)** NPI + Random			
NPI	1.59	1.37, 1.84	<0.001
PLAU	1.29	1.08, 1.54	<0.001
ACTR3C	0.94	0.75, 1.17	0.57
PHKG1	0.74	0.51, 1.07	0.11
RSPRY1	1.46	0.99, 2.15	0.06
P2RX6P	0.91	0.77, 1.06	0.21
TIGD6	1.15	0.75, 1.76	0.52
SPON1	0.78	0.66, 0.92	<0.001
PTPN14	1.44	1.06, 1.97	0.02
PDZK1	0.94	0.87, 1.02	0.12
BTBD10	1.54	0.97, 2.46	0.07
SLC9B2	0.74	0.57, 0.97	0.03
TRIM71	0.79	0.41, 1.49	0.46
ADCY6	0.87	0.62, 1.21	0.40
ALOX15B	0.94	0.84, 1.06	0.34
**(f)** NPI			
NPI	1.65	1.43, 1.90	<0.001

**OncotypeDxGL**. The OncotypeDx signature includes 21 genes associated with the outcome: ESR1 and HER2 along with estrogen-regulated transcripts and proliferation-related genes [33], whose expression values are then combined into a recurrence score that predicts distant recurrence in node-negative, tamoxifen-treated BC. We selected the gene expression list used by this recurrence score. The genes are shown in Table 1c.

Additionally, we included the analysis based on a selection without the housekeeping genes, which were used by [33] for normalization. We denote this additional selection as OncotypeDxRed. The corresponding results can be seen in S2 Appendix.

**Random signatures**. For the sake of simplicity, we randomly selected genes the size of the hybrid signature (15 genes) in the test set 1 as well as in the test set 2. Further, an additional random hybrid set was constructed by substituting one random gene (CADPS2) with the NPI to fully compare it with the hybrid selection. We refer to these selections as Random and NPI+Random signatures, respectively. Both selections can be seen in Table 1d and 1e. Additionally, we generated 100 random signatures and assessed their performance with the statistical measures described below. The corresponding results can be found in S1 Appendix.

### Stratification using decision trees

We used Conditional Inference Decision Trees [34] to find the optimal number of survival risk groups in each signature. Also known as Unbiased Recursive Partitioning, this algorithm recursively partitions and selects dependent variables based on statistical significance, as opposed to the informational gain as is the case by the commonly used decision trees introduced by Breiman et al. [35]. More precisely, variable selection and the exploration of all possible splits for each variable is based on permutation-based significance tests. Similarly, a statistical criterion can be used to decide if the recursion needs to stop, so that no tree pruning is required in contrast to conventional Decision Trees [34].

Concretely, the hazard ratio was considered as the dependent variable in relation to predicted survival time and status. Subsequently, Decision Trees generated the optimal number of nodes, i.e. risk groups, by permuting and splitting hazard ratios according to respective p-values for each signature. The algorithm was implemented using the R package *party* [36].

### Statistical analysis

Different measures can be used to assess the performance of prediction models. However, it is advisable to report not only the overall performance measures but also measures that evaluate calibration as well as discrimination abilities of a prediction model [37, 38].

**Discrimination** describes how well a model splits between risk groups. We compute four commonly used discrimination measures: the Receiver Operating Characteristic (ROC) curve and its integrated area (IAUC), the C-index, and the logrank statistic. The latter is the most popular method for assessing the comparison among risk groups [39].

**Calibration** describes the extent of bias, i.e. how reliable the predicted survival probabilities are. For example, if a model predicts for a similar group of patients a 40% risk, then the observed frequency of dying for this group should be roughly 40%.

**The overall performance** is assessed by calculating the explained variation. This measure quantifies the distance between predicted and observed outcomes. Further we introduce the Signature Skill Score (SSS) as a measure of improvement upon randomly generated signatures. The SSS utilizes the Brier score that combines both discrimination and calibration into a single measure.

#### Discrimination

*AUC and IAUC*. In the standard ROC curve analysis, event status and signature value for an individual are assumed to be fixed over time. This assumption, however, does not reflect the practice, where both the event status and signature values are time-dependent. For example, a disease-free patient may develop the disease later. Thus, the time-dependent approach is more appropriate.

Let *D*_*i*_(*t*) and *X*_*i*_ denote the binary event status at time *t* and the outcome of a signature for patient *i*, respectively. Then, time-dependent sensitivity and specificity can be defined for a given threshold value *c* by

$$\begin{array}{c} Se\left(c,t\right)=P(X_{i}>c|D_{i}\left(t\right)=1),\\ Sp\left(c,t\right)=P(X_{i}\leq c|D_{i}\left(t\right)=0). \end{array}$$

Accordingly, the time-dependent area under the ROC curve (AUC) is defined by

$$\begin{array}{c} AUC\left(t\right)=\int_{-\infty }^{\infty }Se\left(c,t\right)d\left[1-Sp\left(c,t\right)\right], \end{array}$$

which is equal to the probability that a pair of randomly chosen individuals with and without an event are correctly ranked at time *t*. Absent of censoring, AUC and *c*-index are identical. For censored data the time-dependent sensitivity and specificity have to be estimated. For the estimation, we used the method described by Wolf *et al.* [40]. Additionally, IAUCs were computed and tested for differences with the Wilcoxon rank sum test [41] for dependent samples using the R package *survcomp* [42].

*Advantage:* The ROC curve and AUC can be preferred over the C-index to assess the optimal threshold of sensitivity and specificity for a single model.

*Disadvantage:* ROC curves and AUC of censored data can be misleading, since some censored data would have had events if observed for a longer follow-up [37].

*C-index*. The C-index of a model will be large, if patients with longer survival times are scored higher than those with shorter survival times. Strictly, the C-index, also known as Harrell’s index or *c*-statistic, is defined as the probability *P*(*x*_*i*_ > *x*_*j*_ ∣ *y*_*i*_ > *y*_*j*_) of the predicted data *x* agreeing with the observed data *y* [38].

A pair of observations *i*, *j* agree if (*y*_*i*_ > *y*_*j*_, *x*_*i*_ > *x*_*j*_) or (*y*_*i*_ < *y*_*j*_, *x*_*i*_ < *x*_*j*_). The probability can be computed as the fraction of agreed (concordant) pairs:

$$\begin{array}{c} c=\frac{1}{2}(\frac{C-D}{C+D+T_{x}}+1), \end{array}$$

where *C*, *D*, and *T*_*x*_ denote the numbers of the concordant pairs, discordant pairs, and pairs tied to the prediction *x*, respectively. Hereby, ties in *x* score are treated as 1/2 and ties in *y* are treated as incomparable.

*Advantage:* Appropriate for assessing the predictive discrimination of a single model.

*Disadvantage:* The C-index is insensitive for detecting small differences in discrimination between two or more models: As exemplified in [38], The pair (0.01, 0), (0.9, 1) is no more concordant than the pair (0.05, 0), (0.8, 1).

*Logrank test*. The logrank test is widely used to compare the survival of groups [39]. It’s null-hypothesis assumes that there is no difference between groups.

Formally, it is a non-parametric hypothesis test with the null-hypothesis that the groups are sampled from the same population regarding survival experience. Consider *m* groups, then the logrank statistic can be calculated with the following expression:

$$\begin{array}{c} \chi^{2}\left(logrank\right)=\sum_{i=1}^{m}\frac{O_{i}-E_{i}}{E_{i}}, \end{array}$$

where *O*_*i*_ and *E*_*i*_ denote the total number of observed events and the total number of expected (predicted) events in group *i*, respectively.

*Advantage:* No knowledge about the shape of the survival curve or the distribution of survival times is needed.

*Disadvantage:* As a test purely of significance it does not provide an estimate of the difference between the groups.

#### Calibration

To assess the calibration, we plot the observed fraction of survivors against the estimated probability of survival using resampling. Implementation of the plot and details can be found in Frank E Harrell’s R package *rms* [43].

*Advantage:* Calibration plot provides a visual inspection of how accurate the predicted risks are regardless of weather the p-values are significant or not.

*Disadvantage:* Results may differ if other validation techniques such as cross-validation or bootstraping are used [44].

#### Overall performance

*Explained variation.* For the linear regression, the explained variation R^2^ is well defined as the proportion of variance in the dependent variable explained by the model. This definition, however, cannot be applied to categorical and ordinal numbers. Instead, pseudo R^2^ values are computed. We used Nagelkerke’s pseudo R^2^ [45], which is defined as

$$\begin{array}{c} R^{2}=1-\text{exp}\{-\frac{2}{n}(l_{\hat{\beta }}-l_{0})\}, \end{array}$$ (1)

where *n* is sample size, $l_{\hat{\beta }}$ is the maximized log likelihood of the (Cox) model being estimated, and *l*_0_ denotes the log likelihood of null model. In the case of the Cox model, suppose ***x*** is the covariable vector. Then the model being estimated has the linear predictor vector $x\hat{\beta }$, and $2(l_{\hat{\beta }}-l_{0})$ is the likelihood ratio statistic for comparing this model with the null model.

*Advantage:* This measure is consistent with the classical explained variation used in linear regression.

*Disadvantage:* In the presence of censoring, Nagelkerke’s R^2^ is negatively correlated with the proportion of censored data [46], so that low values can be misleading.

*Signature skill score.* Here, we introduce a signature skill score. To calculate the SSS, one needs to compute the Brier score (BS), which combines the calibration and discrimination into a single value. BS is the average mean-squared error [47]:

$$\begin{array}{c} BS=\sum_{i=1}^{m}\frac{\hat{E_{i}}-\hat{O_{i}}}{\hat{E_{i}}}. \end{array}$$

Hereby, $\hat{O_{i}}$ and $\hat{E_{i}}$ denote the actual outcome (1 or 0 for event or non-event without censoring) and the estimated outcomes (survival probabilities) for patient *i*, respectively. The number of patients is denoted by *m*. For censored data the actual outcomes are treated differently. For further details and implementation we refer to Graf *et al.* [44, 48].

In weather forecasting the “so-called” skill score is widely known as the comparison of the model’s Brier score to a reference Brier score [49]: (*BS*_*ref*_ − *BS*)/*BS*_*ref*_. Likewise, SSS can be computed as:

$$\begin{array}{c} SSS=\frac{{\left\langle BS\right\rangle }_{random}^{n}-BS}{{\left\langle BS\right\rangle }_{random}^{n}}, \end{array}$$

where ${\langle BS\rangle }_{random}^{n}$ denotes the mean Brier score of *n* randomly generated signatures. The rationale behind this score is twofold. One should compare the performance of a signature not with the null model of no predictors but with the average performance of many random signatures. On the other hand, a skill score is easy to interpret: An SSS of 0 means that the Brier score of a signature is identical with the Brier score averaged over random signatures and, thus, provides no improvement on just random selections. An SSS of 0.6 would indicate a 60% improvement on random signatures.

*Advantage:* SSS provides an easy to interpret score for comparing the overall performance of a signature with the perfomance of random signatures.

*Disadvantage:* The limitations of SSS are related to those of the Brier score, i.e. one should interpret the scores carefully. Especially without inspection of the calibration plots, one should ask whether the BS is effected by a small number of high errors or a large number of smaller errors.

### Survival analysis

For the METABRIC data, only patients who either died due to the disease or are still alive were taken into account. An event was considered if they died of their disease within 10-year survival time. The time interval between diagnosis and follow-up date was defined as survival time. For the GSE96058, the overall survival time and the overall survival were chosen in correspondence with Brueffer *et al.* [25]. The survival analysis—including the Cox proportional hazards model, estimations of the survival probability, and Kaplan-Meier plots—was performed in the R package *survival* [50]. Below, we use a more stringent convention for statistical significance (p < 0.001 rather than p < 0.05) and refer to this level of significance as “significantly” higher or lower.

### Limitations of our method

The biological processes captured by the Hybrid signature were not addressed, since we approached the prognosis from a systematic data-driven perspective, not from the perspective of systems biology. The use of data from different gene expression microarray platforms might impair the comparison between the competing signatures (see [51] for an estimation of the variability between microarray platforms). Finally, gene lists instead of risk or recurrence scores were taken into account for EndoPredict and OncotypeDx, i.e. expressions of genes that serve as input for their algorithms were considered. This restricts the direct comparison with the corresponding commercially available platforms.

## Results

### Multivariable analysis

Our goal was to compare the inherent prognostic ability of signatures by examining their gene lists. For this matter, we fit the NPI with the univariable Cox model—a model that includes just one variable, namely the NPI. The other signatures were fit with a multivariable Cox model, a model with multiple covariates such as gene expression or prognostic score. The results are shown in Table 1.

### NPI provides more reliable survival probabilities, but their range is limited

**Fig 3:**
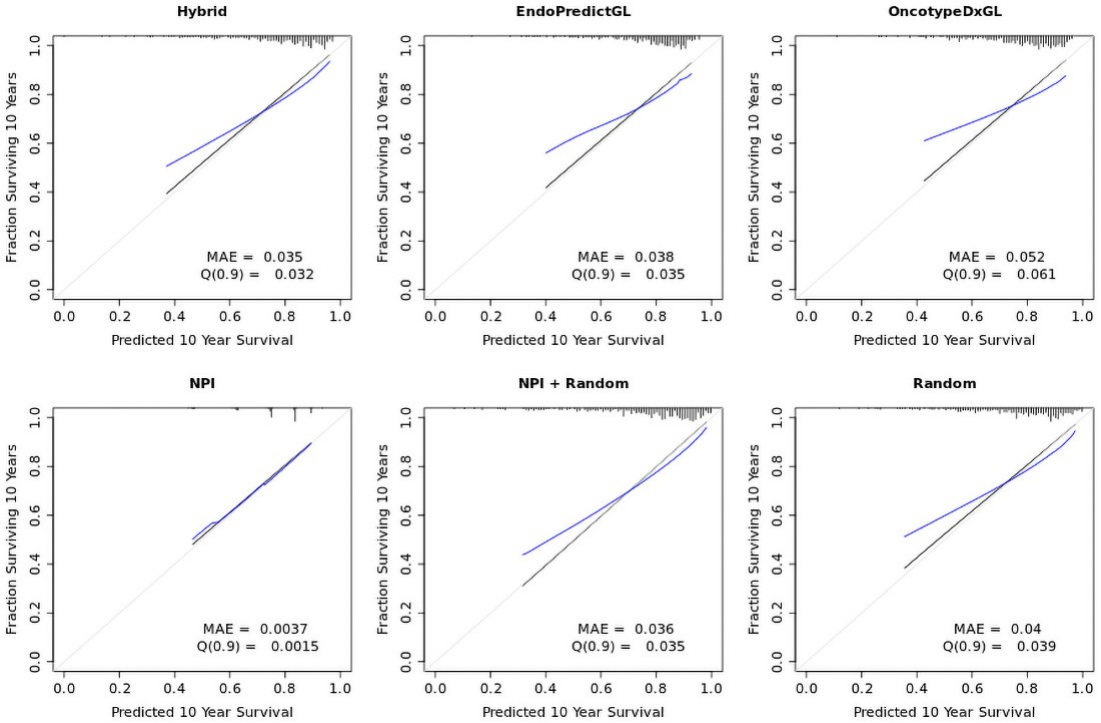
Calibration plots for competing signatures. Observed fraction of survivors (black line) is plotted against the predicted fraction of survivors (blue line) in the test set 1 (METABRIC, n = 379). A perfectly reliable model would show both lines lying on the diagonal (gray line). MAE is the mean absolute error. Q(0.9) is the 0.9 quantile of the MAE, indicating that 90% of errors lie within the interval [0, Q(0.9)].

The question we first addressed was whether the competing signatures were well calibrated, i.e. whether they provided reliable survival probabilities. The calibration for each signature is displayed in Fig 3. Here, the observed proportion of survivors on the y-axis is plotted against the predicted proportion of survivors on the x-axis. A prediction with 100% reliable probabilities would show the observed proportion (black line) and the predicted proportion (blue line) lining on the ideal diagonal (gray line). Consequently, the closer the predicted blue line is to the observed black line, the more reliable are the probabilities predicted by a model. Moreover, the range of predicted probabilities, i.e. the range of the blue line, is also important, since a wider range indicates that a model covers a wider range of patients with different prognosis. For example, the range of probabilities for the NPI shows that the majority of probabilities were predicted and are limited to the range of ca. 0.5–0.9. This was reflected in the Kaplan-Meier plots in Fig 5 and in Fig 6.

To quantify the distance between the predicted blue line and the observed black line, the mean absolute error (MAE) was computed. From the calibration plots we first see that the other signatures cover a slightly wider range of survival probabilities (ca. 0.4–1.0) than the NPI. If we neglect the difference in probability ranges, one can see that the NPI provided more reliable survival probabilities (MAE = 0.0037) than other signatures. However, it is advisable to compare the MAE only between these signatures. As seen in Fig 3, the MAE was the lowest for the Hybrid signature (0.035) and NPI+Random signature (0.036), followed by EndoPredictGL (0.038), Random (0.040), and OncotypeDxGL (0.052).

All signatures showed comparable calibrations, overestimating the proportions of patients with bad prognosis and slightly underestimating the proportions with good prognosis. However, the rug plot (top marks along the x-axis) of the NPI showed that—in contrast to other signatures—its survival probabilities were not equally distributed along the x-axis but were rather grouped across four centers, indicating that the NPI provided a rough classification of patients. This was again reflected in the Kaplan-Meier plots in Fig 5 and in Fig 6.

Considering the calibrations, our results suggest that:

The NPI provides a more reliable prognosis, but their range of reliable prognoses is limited. For example, no assessment could be done for patients who would definitely die or survive. Other signatures covered a wider range of prognosis than the NPI, but their reliability was smaller. They also showed comparable results, even including the Random signature.

### None of the competing signatures demonstrate substantially higher IAUCs

**Fig 4:**
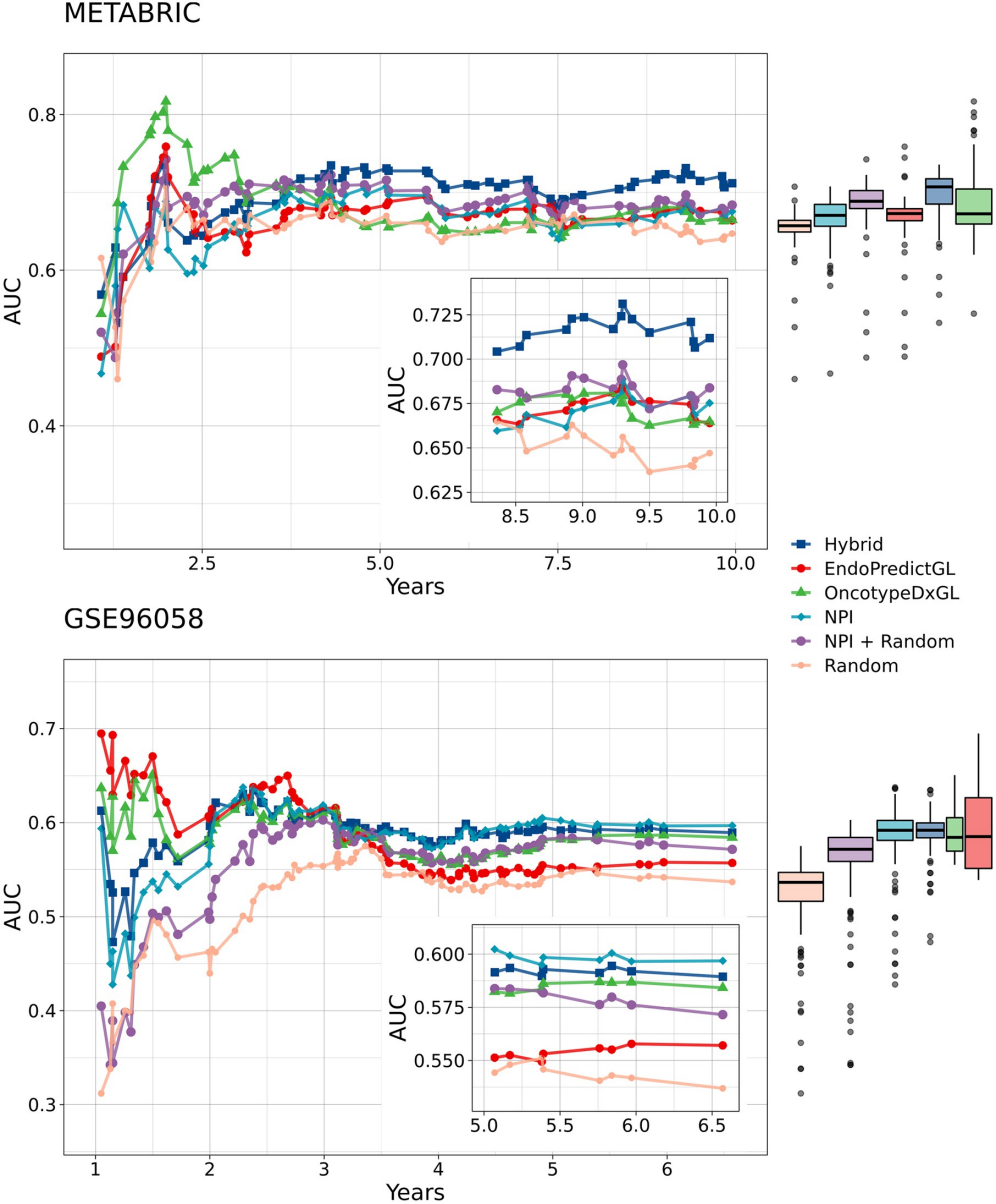
Time-dependent area under the curve (AUC) of competing signatures. (top) Time-dependent AUC of competing signatures for patients in the test set 1 (METABRIC, n = 379). (bottom) Time-dependent AUC of competing signatures for patients in the test set 2 (GSE96058, n = 440). The insets show AUCs within the last 1.5 observation years. In the marginal plots the corresponding boxplots are shown.

To investigate the prognostic ability of each signature in more detail, we depicted the time-dependence of AUC over observation time in Fig 4. Please remember: the AUC is equal to the probability that a pair of randomly chosen individuals with and without an event are correctly ranked at time *t*. Here, the results for both test sets METABRIC and GSE96058 are shown. As displayed in the figure, AUC values fluctuated initially for all signatures and for both data sets in the first 3 years, exhibited slight fluctuations between years 3 and 7.5 for the test set 1 and between years 3 and 5 for the test set 2, until they equilibrated in the last 1.5 years. These fluctuations may be attributed simply to the fact that for this short period of time (< 3 years) non-event data were incorrectly classified, since for the longer follow-up they would have had events. For example, the first time point for the classification was at 0.46 years, i.e. roughly 6 months after surgery. Thus, a patient who died 12 months thereafter was incorrectly classified as non-event at 0.46 years. Another contributing factor is that the Cox models were fitted based on endpoints that are longer than 3 years.

For the test set 1, the Hybrid signature showed the highest AUC values in the last 1.5 years whereas the Random signature exhibited the lowest values. The other signatures differed only slightly, which can be inspected in the inset of top Fig 4. The corresponding marginal boxplots in top Fig 4, nevertheless, indicate that globally the AUCs were distributed across a comparable range for all signatures. For the test 2, the NPI showed the highest AUC values in the last 1.5 years whereas the Random signature exhibited again the lowest values. Except the EndoPredictGL signature other signatures differed only slightly, which can be inspected in the inset of bottom Fig 4. The corresponding marginal boxplots in bottom Fig 4 indicate anew that globally the AUCs were distributed across a comparable range for all signatures except the Random signature.

On the basis of AUCs shown in Fig 4 we calculated IAUCs and examined if the differences between IAUCs were statistically significant by performing the Wilcoxon rank sum test for dependent samples. Table 2 shows the results. As seen, the Hybrid, OncotypeDxGL, and Random signatures demonstrated consistent results in both data sets. Specifically, the Hybrid signature showed the highest IAUCs that were significant in both data sets. The OncotypeDxGl signature showed the second highest IAUCs but its p-values were not significant in the test set 2. Moreover, except the NPI+Random and Random signatures all signatures had the same IAUC of 0.60 which differed in the third decimal place. The Random signature showed the lowest IAUCs that were statistically non-significant in both data sets.

**Table 2:** Test of differences between IAUCs calculated on AUCs shown in Fig 4. P-values from the Wilcoxon rank sum test for dependent samples. P-values are computed for the same points in time for the comparison IAUC1 > IAUC2, where IAUC1 denotes the IAUC in a row and IAUC2 denotes the IAUC in a column. For both data sets, rows are sorted w.r.t. the corresponding IAUC values in descending order (For the test set 1: Hybrid > OncotypeDxGL > NPI+Random > …).

**Test set 1 (METABRIC)**							
	**IAUC**	**Hybrid**	**EndoPredictGL**	**OncotypeDxGL**	**NPI**	**NPI + Random**	**Random**
**Hybrid**	0.691	-	<0.001	0.037	<0.001	<0.001	<0.001
**OncotypeDxGL**	0.679	0.969	0.023	-	0.049	0.972	<0.001
**NPI + Random**	0.664	0.999	<0.001	0.029	<0.001	-	<0.001
**NPI**	0.657	1	0.433	0.951	-	1	<0.001
**EndoPredictGL**	0.650	1	-	0.977	0.568	1	<0.001
**Random**	0.640	1	0.999	1	0.999	1	-
**Test set 2 (GSE96058)**							
	**IAUC**	**Hybrid**	**EndoPredictGL**	**OncotypeDxGL**	**NPI**	**NPI + Random**	**Random**
**Hybrid**	0.608	-	0.042	<0.001	0.027	<0.001	<0.001
**OncotypeDxGL**	0.604	0.999	0.531	-	0.991	<0.001	<0.001
**EndoPredictGL**	0.603	0.957	-	0.471	0.869	0.008	<0.001
**NPI**	0.602	0.974	0.131	0.009	-	<0.001	<0.001
**NPI + Random**	0.541	1	0.993	1	1	-	<0.001
**Random**	0.491	1	1	1	1	1	-

Considering the AUCs and IAUCs, our results suggest: None of the competing signatures seems to exhibit superior performance, since the high IAUC of the Hybrid signature in the test set 1 can be attributed to the fact that this data set is sampled from the same data distribution as the training data set, on which the Hybrid signature was developed. Only the Random signature consistently shows the lowest discrimination ability.

### None of the competing signatures shows substantially higher discrimination ability and superior overall performance

Please recall that the IAUC—which is also shown in Table 3 for comparison purposes—reflects the discrimination ability of a prediction model as a summary index for the binary outcome. However, the C-index describes the discrimination ability of a prediction model as well by extending AUC to censored data and R^2^ quantifies the goodness of fit.

**Table 3:** Overall performance of competing signatures. The Signature Skill Score (SSS) was computed using 100 random signatures that were generated additionally and did not contain Random and NPI+Random signatures.

	C-index	IAUC	SSS	Nagelkerke’s R^2^
**Training set (METABRIC)**				
**Hybrid Signature**	0.745	0.775	0.132	0.163
**EndoPredictGL**	0.679	0.691	0.072	0.094
**OncotypeDxGL**	0.724	0.729	0.084	0.128
**NPI**	0.678	0.701	0.024	0.057
**NPI + Random**	0.692	0.697	0.049	0.096
**Random**	0.631	0.620	0.013	0.053
**Test set 1 (METABRIC)**				
**Hybrid Signature**	0.690	0.691	0.000	-
**EndoPredictGL**	0.652	0.679	-0.011	-
**OncotypeDxGL**	0.652	0.664	-0.011	-
**NPI**	0.658	0.657	0.037	-
**NPI + Random**	0.668	0.650	-0.049	-
**Random**	0.637	0.640	-0.037	-
**Test set 2 (GSE96058)**				
**Hybrid Signature**	0.615	0.608	0.009	-
**EndoPredictGL**	0.572	0.604	-0.028	-
**OncotypeDxGL**	0.592	0.603	-0.028	-
**NPI**	0.614	0.602	-0.009	-
**NPI + Random**	0.597	0.541	-0.009	-
**Random**	0.554	0.491	-0.019	-

Table 3 illustrates that the Hybrid signature showed the highest values for the C-index and the Nagelkerke’s R^2^. In regard of these results, the Hybrid signature seems to demonstrate higher discrimination ability and higher overall performance. On the other hand, Table 3 shows that the C-Index of the Hybrid signature (0.615) is only slightly higher than the C-index of the NPI (0.614) in the test set 2. Moreover, the C-indices were consistent only for the Hybrid and the Random signatures in both data sets.

To clarify these discrepancies, we computed the signature skill score, which quantified the improvement of competing signatures on the mean of 100 signatures selected at random. These 100 random signatures were generated additionally and did not include the Random or NPI+Random signatures. As demonstrated in Table 3, the NPI showed the highest SSS in the test set 1 and made 3.7% improvement, whereas other signatures showed no improvement or even a negative SSS, i.e. a decrease in skill. On the other hand, these results could not be confirmed in the test set 2, since only the Hybrid signature showed a positive SSS of 0.009, whereas other signatures indicated a decrease in skill.

These results showed that: The Hybrid signature indicated a slightly higher discrimination ability and a higher overall performance than other signatures. Nonetheless, none of the signatures shows a substantially superior performance, which is also supported by results of repeating the downsampling 1000 times (see S2 Appendix), and none of the signatures shows a substantial improvement upon random signatures.

### Decision Trees suggest that Hybrid and OncotypeDxGL signatures can better guide decision to omit chemotherapy than competing signatures

**Fig 5:**
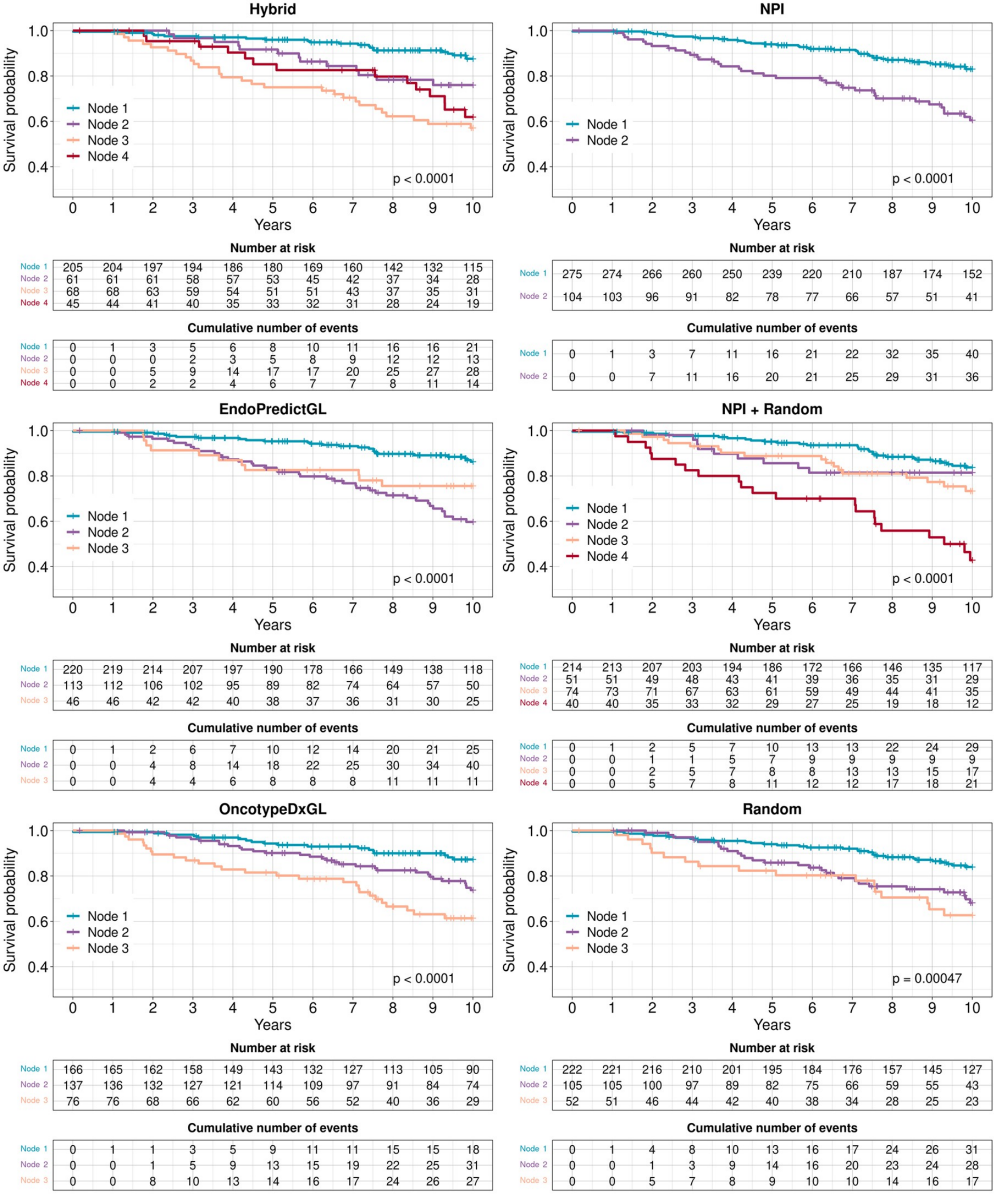
Survival curves of no chemo patients in the test set 1 (METABRIC) with respect to risk classifications for each signature. Risk groups were identified by Decision Trees. For each signature, the algorithm found different numbers of risk groups indicated by Node 1, Node 2, etc. P-values were calculated from the two-sided logrank test.

**Fig 6:**
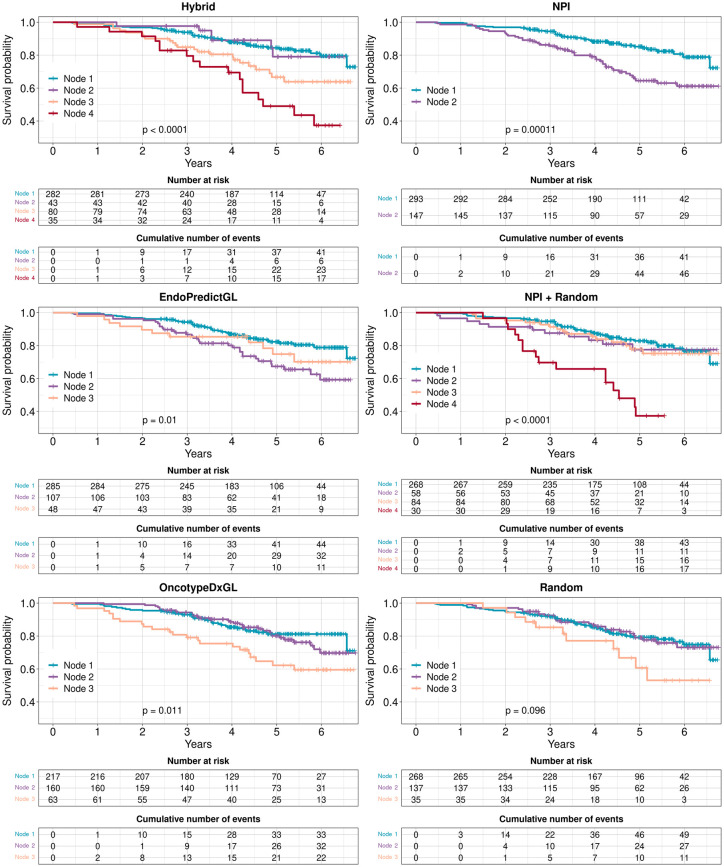
Survival curves of no chemo patients in the test set 2 (GSE96058) with respect to risk classifications for each signature. Risk groups were identified by Decision Trees. For each signature, the algorithm found different numbers of risk groups indicated by Node 1, Node 2, etc. P-values were calculated from the two-sided logrank test.

In order to stratify patients universally for all signatures, we applied Decision Trees to hazard ratios of each model. Survival curves of the risk groups resulting from Decision Trees are displayed in Figs 5 and 6 for the test set 1 and test set 2, respectively. Notably, the number of risk groups, i.e. the optimal number of nodes, was computed by Decision Trees. In regards to p-values, survival curves were significantly different between risk groups for all six signatures. Still, to evaluate the Kaplan-Meier curves precisely, we considered the distribution of events and numbers at risk as well as the number of risk groups for each signature.

In this context, the NPI and the Random signature showed the lowest performances: The NPI divided patients into only 2 risk groups in both data sets. Although the Random signature divided patients into 3 risk groups, its Node 2 and Node 3 risk groups in the test set 1 as well as its Node 1 and Node 2 in the test set 2 overlap and could be united to a single risk group, respectively.

The remaining candidates for the best performance were the Hybrid, OncotypeDxGL, EndoPredictGL, and NPI+Random signatures. Hybrid and NPI+Random had 4 risk groups but EndoPredictGL and OncotypeDxGL only 3 groups. However, in Figs 5 and 6 one can see that the risk groups of these signatures intersected and overlapped in both data sets, indicating that 3 risk groups would have been more representative. The risk groups of EndoPredictGL and OncotypeDxGL signatures intersected or overlapped as well, but this was not representative in both data sets.

**Fig 7:**
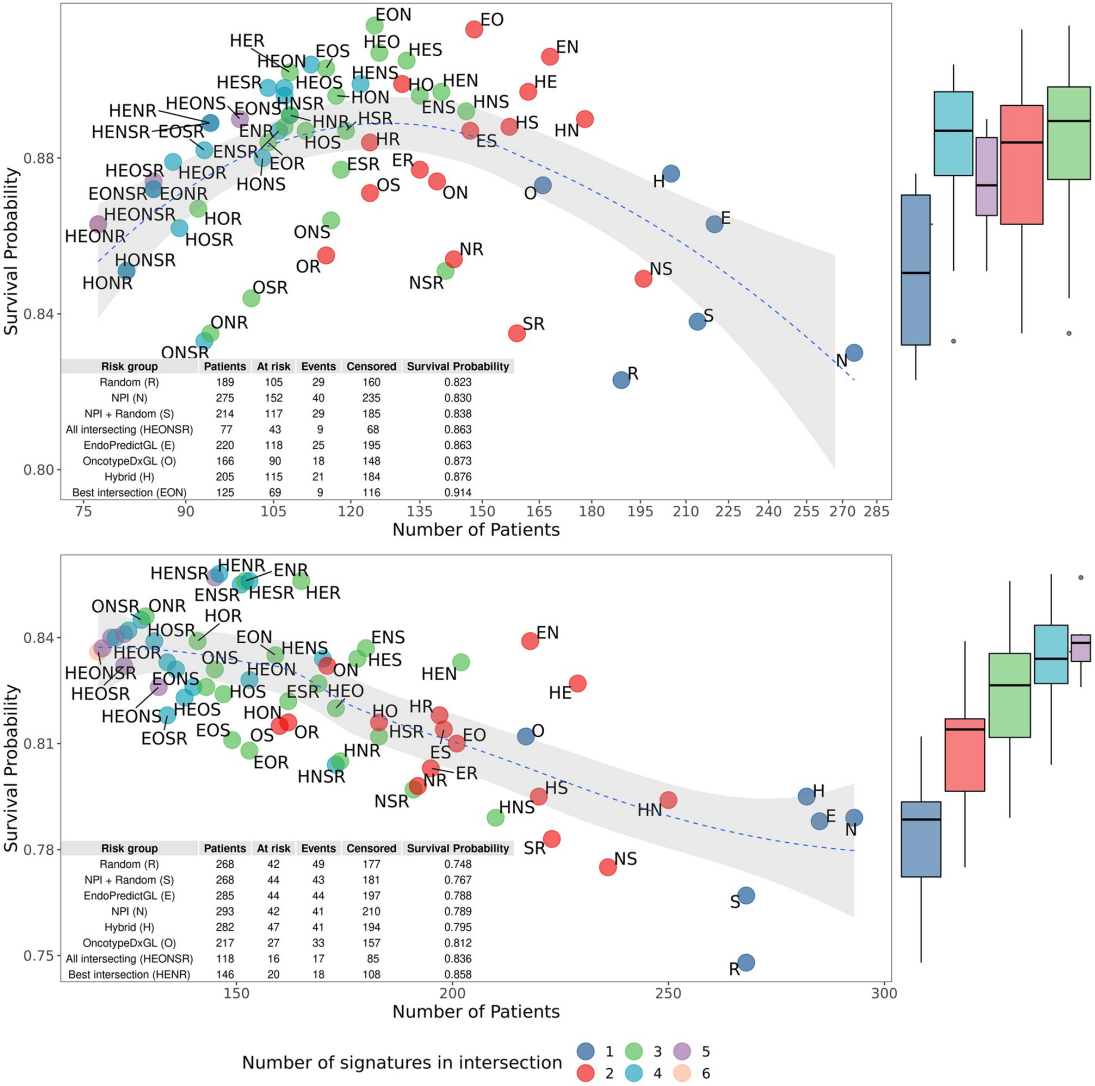
Survival probabilities for different combinations of groups with the best prognosis. (top) Test set 1 (METABRIC, n = 379). (bottom) Test set 2 (GSE96058, n = 440). Patients only in the Node 1 risk group shown in Fig 5 and in Fig 6 are selected, i.e. risk groups with the best prognosis. We identify intersections: those patients who are shared between these Node 1 risk groups, and compute the corresponding survival probabilities. “Number of signatures in intersection” means the number of signatures sharing this particular intersection. For example, HEO is shared by 3 signatures, namely by the Hybrid (H), EndoPredictGL (E), and OncotypeDxGL (O) signatures. Survival probabilities are based on the Kaplan-Meier estimates for 10-year disease-free survival for the test set 1 and for the overall survival (provided in GSE96058) for the test set 2. Please note that each plot has different ranges of the number of patients on the x-axis.

In order to guide decisions to omit chemotherapy, however, one has to take the relative survival probability of the best group and its size into account, since the survival probability of a group with a very high probability but a small number of patients may not be reliable. The relative survival probability for each signature can be seen in the inset tables of Fig 7. For the test set 1, the according values in decreasing order were 0.876, 0.873, 0.863, and 0.838 for the Hybrid (205 patients), OncotypeDxGL (166 patients), EndoPredictGL (220 patients), and NPI+Random (214 patients) signatures, respectively. For the test set 2, the according values in decreasing order were 0.812, 0.795, 0.788, and 0.767 for the OncotypeDxGL (217 patients), Hybrid (282 patients), EndoPredictGL (285 patients), and NPI+Random (268 patients) signatures, respectively. Consequently, the OncotypeDxGL showed the second highest and the highest relative survival probability of its best risk group in the test set 1 and test set 2, respectively. However, it also had the lowest number of patients compared to other signatures in both data sets.

Two conclusions may be drawn from these results. First, the Hybrid, OncotypeDxGL, and EndoPredictGL signatures showed best and roughly equal risk stratification. Second, the guidance for chemotherapy is more reliable based on the Hybrid and OncotypeDxGL signatures.

### Taking the results of multiple signatures into account can improve guidance for chemotherapy omission

We investigated if one can improve guidance for chemotherapy omission by taking the predictions of multiple signatures into account. For this purpose, we selected patients only in the Node 1 risk group of all signatures, i.e. risk groups with the best prognosis. We then identified which patients were shared between all possible combinations of these risk groups, and computed the corresponding relative survival probabilities. For example, we identified which patients were assessed less dangerous in both the Hybrid signature and the NPI, which patients were assessed less dangerous in the Random and OncotypeDxGL as well as EndoPredictGL signatures, etc. The results of these identifications can be seen in Fig 7.

The abbreviation HR represents patients with the best prognosis who were shared by the Hybrid (H) and the Random (R) signatures; and ENS includes those patients who were shared by the EndoPredictGL (E) signature, the NPI (N), and the NPI+Random (S) signature, and so on. In this figure, the survival probability is plotted against the number of shared patients. Further, we color-coded the number of signatures used to identify shared patients, i.e. the number of signature “tests”. From this, one can observe that additional tests (from right to left in Fig 7) continuously increased the survival probability in the test set 2. The survival probability increased in the test set 1 as well but decreased again after the number of shared patients reduced to ca. 130, indicating that with less than 130 patients the shared risk groups were not anymore representative. The inset tables of Fig 7 show the characteristics for selected risk groups. Here, we also show results for those patients who received the best prognosis in all six signatures (“All intersecting”) and for patients with the overall highest survival probability (“Best intersection”). From here and from the marginal boxplots for both data sets, one can clearly see that taking the results of multiple signatures tended to improve the survival probability and, thus, better guide the decision to omit chemotherapy.

The increased survival rates for adding multiple signature tests suggest that the signatures may misidentify different patients, i.e. they make errors on *different*—not the same—patients, since otherwise all signatures would have shown the same predictions and no increase in survival probability.

**Fig 8:**
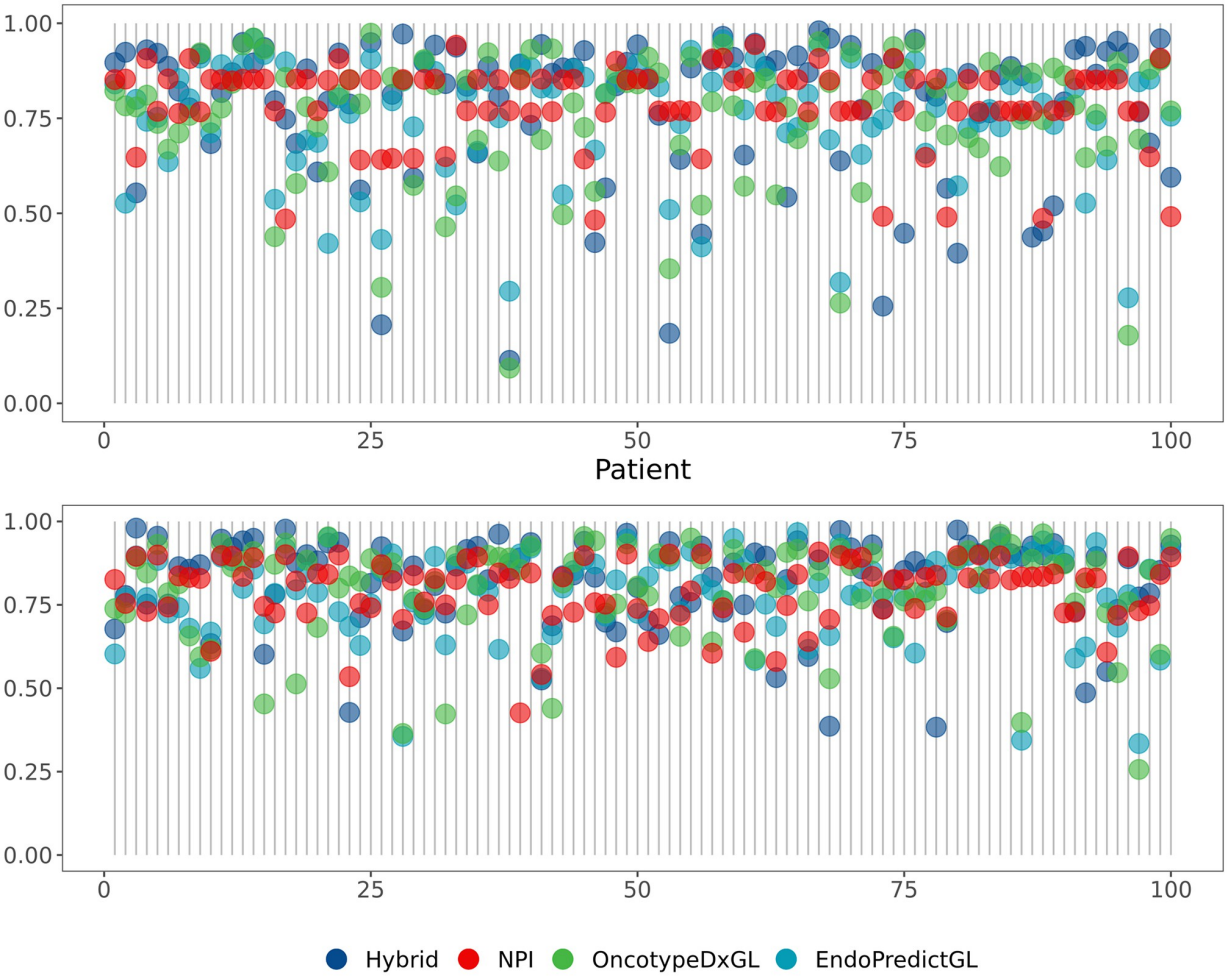
Predicted survival probability for four competing signatures. (top) 100 patients selected at random in the test set 1 (METABRIC). (bottom) 100 patients selected at random in the test set 2 (GSE96058). A gray line represents a single patient on the x-axis. A perfect agreement between the signatures would show all points completely overlapping. The probabilities are based on the 10-year disease-free survival in the test set 1 and on the overall survival (provided in GSE96058) for the test set 2.

To validate this hypothesis, we selected 100 patients at random for each data set and plotted in Fig 8 the predicted survival probabilities (y-axis) for each patient (x-axis). Here, we concentrated on Hybrid, EndoPredictGL, OncotypeDxGL, and NPI, since the latter 3 signatures are used in routine clinical practice. If all 4 signatures would agree for all patients, the points would perfectly overlap. However, we see a wide spread of disagreement for almost every patient in both data sets. Especially regarding the patients with bad prognosis, the signatures seem to disagree at most.

## Discussion

We aimed to find a signature that substantially improves prediction by introducing a hybrid approach. In this connection, we did not aim to infer biological meaning from the Hybrid signature. On the contrary, our study implies that inferring biological interpretation from significant associations with survival outcome might be unjustified, since even a pair of random gene selections can show similar if not identical results. While the gene selection varies dependent on the patients used in the training set for the Hybrid signature, there is no substitute for the NPI, i.e. it is always preferred to gene expression data.

Both findings indicate that it is not sufficient to judge a signature based on the statistical significance of dependent variables in the Cox model. One should rather introduce a valid baseline such as a simple comparison with one or more random signatures on the basis of all measured statistics.

Importantly, the association of Random signatures with outcome does not necessarily imply that the other signatures are not useful. On the contrary, this indicates that even a model based on a randomly chosen set of genes contains prognostically relevant information. It should also be noted that random signatures are predictive, since more than 50% of the breast cancer transcriptome is correlated with proliferation, which incorporates prognostic information, as reported in [14].

Further, our statistical measures demonstrate that even a random set of genes is highly associated with BC outcome even if one uses robust measures such as time dependent AUC and IAUC. This confirms findings of Venet *et al.* [14].

One can argue that the use of gene lists may not be a valid comparison for the two commercially available signatures, since they contain highly correlated gene expressions due to functional relation. However, our aim was to compare the inherent power of genes contained in each signature. Nonetheless, we evaluated the presence of multicollinearity by computing the variance inflation factor (see S3 Appendix): none of these signatures shows a large amount of multicollinearity. It is noteworthy, however, that the OncotypeDx signature was developed on and for lymph node negative breast cancers, and thus, may not be favorable for our patients selection.

In regards to calibration, AUCs and IAUCs, C-Indices, and survival curves, no clear conclusion can be drawn whether the Hybrid signature performs substantially better than other signatures. All presented signatures seem to fluctuate around the AUC of 0.66 got the test set 1 and around 0.60 for the test set 2, and the Signature Skill Scores of all signatures seem to add 0% improvement. We only see a slight improvement for the Hybrid signature in terms of discrimination and benefit from chemotherapy, which stresses not so much this particular signature selection but rather the benefits of a general hybrid approach. Generally, this strongly indicates that combining clinical and pathological characteristics with molecular data in a data-driven approach presents a valuable method to improve patient prognosis. As can be seen in S4 Appendix, this conclusion is further supported if we combine the NPI score with the signature score in a bivariable model, since these bivariable models show slightly better results than the corresponding Hybrid and NPI+Random signatures.

Further, adding random genes to the NPI in the NPI+Random signature facilitated a splitting of a risk group with bad prognosis that could not be found using the NPI alone, as we saw it in the survival curves for both test datasets. This points to an interesting direction that need to be further investigated. This also substantiates that the NPI remains a powerful prognostic score and can be supported by means of molecular approaches.

As indicated in later sections of our study, conducting multiple signature tests can improve prediction for chemotherapy benefit. The authors of [52] showed that combining gene signatures improves prediction of breast cancer survival by using the principal component analysis to derive covariables for the Cox model. To the best of our knowledge, however, it is the first time that a combining of the best groups from multiple signature tests has been done. Our rationale is simple: if two or more signatures identify same patients, there would be no improvement. Thus, if combining of multiple signatures shows an improvement of prediction, than the signatures will misidentify different patients, which we showed in Fig 8.

## Conclusions

The great promise of precision oncology is to predict patient outcome and identify therapy targets—relying on genetic databases such as the Cancer Genome Atlas or METABRIC, independently of tumor histology. Although our study shows the usefulness of analyzing such databases, it also points at limitations of a molecular approach: rather than excluding tumor histology or more generally clinical and pathological characteristics, precision oncology should take such characteristics into account.

Moreover, our results support the idea that there is no universal signature. To put this idea in perspective: If we aim to select 15 genes from 24,368 genes in the MB dataset, then there are 24, 368!/(24, 353!15!) = 4.829775710492069^53^ possible combinations, which is comparable with the lower bound of the possibly infinite diameter of the universe measured in meters [53].

The large variation of genes used in different signatures points at several problems of molecular characterization. The first problem is the loss of spatial information critical to understand cell interactions, which is also tightly related to the problem of the host-tumor interaction. For example, gene signatures are generally derived from whole tissue excluding samples with low tumor cell content. As a result, interactions with components of tumor micro-environment such as tumor-associated stromal cells and infiltrating immune cells are often insufficiently considered.

Signatures based on genes involved in tumor- and immune interactions may provide more accurate results. For instance, Teschendorff *et al.* reported a signature related to immune response and significantly associated with the risk of distant metastasis in ER negative patients [54].

Together, our findings underline that breast cancer manifests itself in many dimensions of interactions such as cell-cell, spatial, host-tumor interactions, which in turn are highly complex in themselves.

In this light, we would like to convey to the medical community the importance of a hybrid approach where clinicians do not rely on a single signature, but consider both the clinical and pathological characteristics as well as molecular signatures.

## Supporting information

S1 Appendix Additional random signatures. (PDF)

S2 Appendix Downsampling. (PDF)

S3 Appendix Multicollinearity [55]. (PDF)

S4 Appendix Bivariable models. (PDF)

S1 Table Descriptive statistics of selected METABRIC data. (PDF)

S2 Table Descriptive statistics of selected GSE data. (PDF)
