# Supplementary material for: Comparative analysis of molecular signatures reveals a hybrid approach in breast cancer: Combining the Nottingham Prognostic Index with gene expressions into a hybrid signature
Source: PLoS One. 2022 Feb 10;17(2):e0261035. doi: 10.1371/journal.pone.0261035 (PMC8830616; doi:10.1371/journal.pone.0261035)
Supplement: S1 Appendix — (PDF) [file pone.0261035.s001.pdf]

S1 Appendix. Additional random signatures

For both the test set 1 and test set 2 we generated 100 additional random signatures and computed AUCs over time that are shown in Fig. 9. Further, histograms of the C-index, Nagelkerke's R^2, Brier score, and LogRank statistic are shown in Fig. 10.

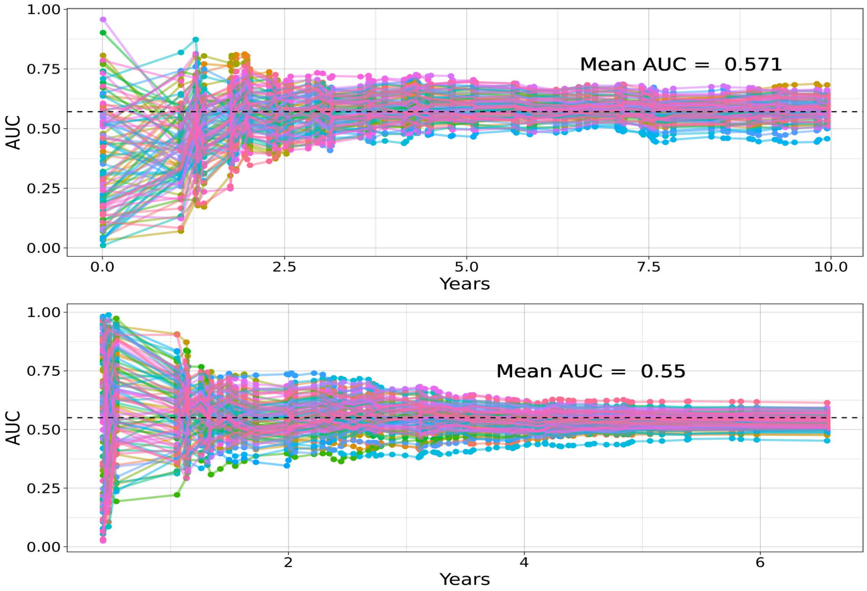

Figure 9. Time-dependent area under the curve (AUC) of additional 100 random signatures (top) Test set 1 (METABRIC). (bottom) Test set 2 (GSE96058). The horizontal blue line represents the overall mean of AUCs.

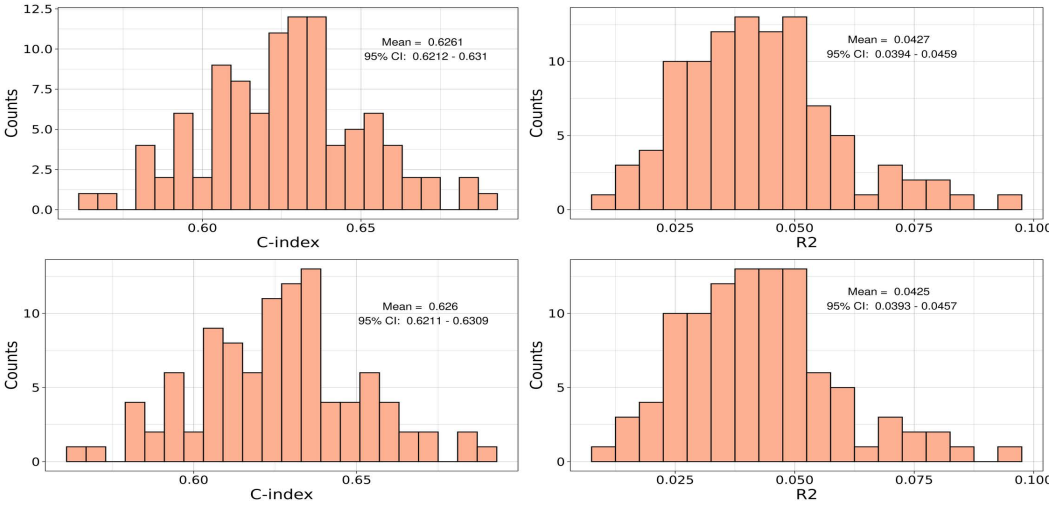

Figure 10. Histograms of C-index and Nagelkerke's R^2 for 100 signatures generated at random. (top) Test set 1 (METABRIC). (bottom) Test set 2 (GSE96058).
