# Supplementary material for: Comparative analysis of molecular signatures reveals a hybrid approach in breast cancer: Combining the Nottingham Prognostic Index with gene expressions into a hybrid signature
Source: PLoS One. 2022 Feb 10;17(2):e0261035. doi: 10.1371/journal.pone.0261035 (PMC8830616; doi:10.1371/journal.pone.0261035)
Supplement: S2 Appendix — (PDF) [file pone.0261035.s002.pdf]

S2 Appendix. Downsampling

The downsampling procedure was repeated 1000 times in the GSE96058 dataset. We also included the gene list used for OncotypeDxGL signature without the 5 housekeeping genes. This reduced signature is denoted as OncotypeDxGLRed. The results of the Cox analysis for this signature are listed in Table 4. The AUCs of the downsampling procedures are shown in Fig. 11 and the overall performance can be seen in Table 5.

Table 4. Cox Proportional Analysis of the OncotypeDxGLRed signature in the METABRIC training set (n = 883). The 1st column lists gene names used in the signature. HR: Hazard Ratio, CI: Confidence Interval.

	HR	CI.Lower	CI.Upper	p-value
MKI67	1.20	0.94	1.54	0.14
AURKA	0.99	0.72	1.35	0.93
BIRC5	1.43	1.10	1.86	0.01
CCNB1	1.18	0.91	1.54	0.21
MYBL2	0.91	0.79	1.04	0.17
ERBB2	0.58	0.40	0.85	0.01
GRB7	1.39	0.88	2.20	0.16
ESR1	0.89	0.77	1.03	0.13
PGR	0.82	0.75	0.90	< 0.001
BCL2	1.03	0.82	1.29	0.81
SCUBE2	1.10	1.01	1.21	0.04
CTSV	1.10	0.86	1.42	0.43
MMP11	1.09	0.96	1.23	0.18
BAG1	0.73	0.55	0.98	0.03
CD68	0.90	0.69	1.17	0.43
GSTM1	0.95	0.91	1.01	0.08

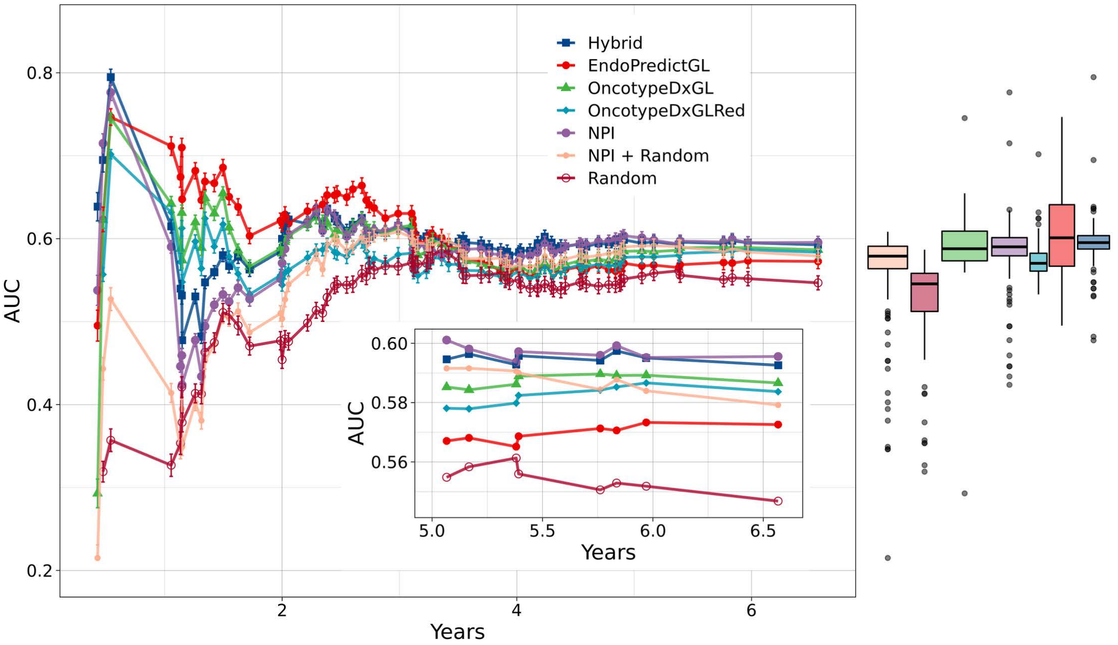

Figure 11. Time-dependent area under the curve (AUC) of 1000 downsampled test sets in the GSE96058 dataset.

Table 5. Overall performance of competing signatures from the downsampling procedure. The means, the upper 95% confidence interval as well as the lower 95% confidence interval for C-Index, IAUC, and SSS are displayed. The Signature Skill Score (SSS) was computed using 100 random signatures that were generated additionally and did not contain Random and NPI+Random signatures.

	C-Index	95%CI		IAUC	95%CI		SSS	95%CI	
Hybrid	0.6178	0.6172	0.6183	0.5999	0.5995	0.6003	0.0107	0.0097	0.0116
EndoPredictGL	0.5935	0.5929	0.5941	0.6018	0.6013	0.6023	-0.0036	-0.0044	-0.0028
OncotypeDxGL	0.5987	0.5981	0.5992	0.5771	0.5766	0.5775	0.0156	0.0148	0.0164
OncotypeDxGLRed	0.5899	0.5893	0.5904	0.5533	0.5528	0.5537	0.0168	0.0161	0.0175
NPI	0.6155	0.6150	0.6160	0.5837	0.5833	0.5841	0.0226	0.0219	0.0233
NPI + Random	0.6089	0.6083	0.6094	0.5279	0.5274	0.5283	0.0244	0.0237	0.0251
Random	0.5690	0.5684	0.5696	0.4880	0.4875	0.4885	-0.0018	-0.0025	-0.0011
