# Supplementary material for: Comparative analysis of molecular signatures reveals a hybrid approach in breast cancer: Combining the Nottingham Prognostic Index with gene expressions into a hybrid signature
Source: PLoS One. 2022 Feb 10;17(2):e0261035. doi: 10.1371/journal.pone.0261035 (PMC8830616; doi:10.1371/journal.pone.0261035)
Supplement: S3 Appendix — (PDF) [file pone.0261035.s003.pdf]

S3 Appendix Multicollinearity

To evaluate the multicollinearity in the gene lists of two commercially available signatures, we computed the variance inflation factor (VIF) for these signatures (see Table 6). The VIF measures the inflation of multicollinearity in the variance of a regression coefficient [55]. A VIF larger than 5 indicates a problematic presence of multicollinearity [55]. As one can see, none of the genes exceeds this value so that none of these signatures seems to exhibit a problematic amount of multicollinearity.

Table 6. Variance Inflation Factor (VIF) for the EndoPredictGL, OncotypeDxGL, and OncotypeDxGLRed signatures.

(a) EndoPredictGL

	VIF
ESR1	1.33
ERBB2	1.26
BIRC5	2.92
RBBP8	1.26
UBE2C	3.26
IL6ST	1.62
AZGP1	1.47
DHCR7	1.48
MGP	1.24
STC2	1.45
CALM2	2.10
PPIA	2.40
OAZ1	1.12
RPL37A	2.46
PAEP	1.04

(b) OncotypeDxGL

	VIF
MKI67	1.90
AURKA	3.53
BIRC5	3.32
CCNB1	2.08
MYBL2	1.07
ERBB2	2.15
GRB7	2.08
ESR1	1.50
PGR	1.17
BCL2	1.66
SCUBE2	1.42
CTSV	1.57
MMP11	1.34
BAG1	1.26
CD68	1.32
GSTM1	1.08
ACTB	1.45
GUSB	1.28
GAPDH	1.45
RPLP0	1.70
TFRC	1.41

(c) OncotypeDxGLRed

	VIF
MKI67	1.80
AURKA	3.31
BIRC5	3.18
CCNB1	1.78
MYBL2	1.04
ERBB2	2.05
GRB7	2.11
ESR1	1.40
PGR	1.13
BCL2	1.51
SCUBE2	1.36
CTSV	1.51
MMP11	1.19
BAG1	1.24
CD68	1.35
GSTM1	1.02
