# Supplementary material for: Comparative analysis of molecular signatures reveals a hybrid approach in breast cancer: Combining the Nottingham Prognostic Index with gene expressions into a hybrid signature
Source: PLoS One. 2022 Feb 10;17(2):e0261035. doi: 10.1371/journal.pone.0261035 (PMC8830616; doi:10.1371/journal.pone.0261035)
Supplement: S4 Appendix — (PDF) [file pone.0261035.s004.pdf]

S4 Appendix Bivariable Models

We also combined the NPI score with the signature score in a bivariable Cox model (Figs. 12, 13, 14, Table 7 and Table 8). In other words, only the gene expression list without the NPI was used to compute the signature score, which was then used in a bivariable model to build a new Cox model. This was conducted for the Hybrid signature and the NPI + Random signature. We denote these bivariable models by NPI + Developed Signature Score and NPI + Random Signature Score, respectively.

Table 7. Cox Proportional Analysis of bivariable models in the METABRIC training set. The 1st column lists the covariables used in the Cox regression . NPI: Nottingham Prognostic Index, HR: Hazard Ratio, CI: Confidence Interval.

(a) NPI + Developed Signature Score

	**HR**	**95% CI**	**p-value**
Score	2.46	2.05, 2.96	< 0.001
NPI	1.50	1.29, 1.75	< 0.001

(b) NPI + Random Signature Score

	**HR**	**95% CI**	**p-value**
Score	1.56	1.35, 1.80	< 0.001
NPI	1.56	1.35, 1.80	< 0.001

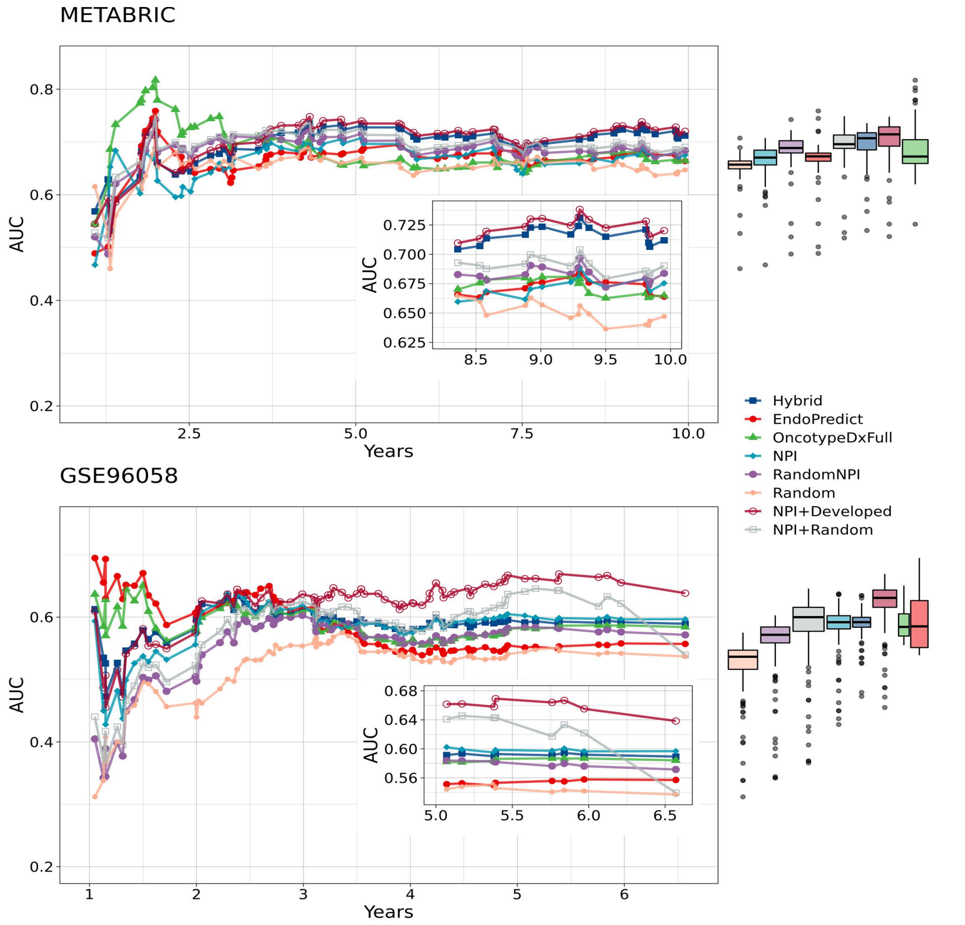

Figure 12. Time-dependent area under the curve (AUC) of bivariable models in comparison to other signatures. (top) Time-dependent AUC of bivariable models for patients in the test set 1 (METABRIC, n = 379). (bottom) Time-dependent AUC of bivariable models for patients in the test set 2 (GSE96058, n = 440). The insets show AUCs within the last 1.5 observation years. In the marginal plots the corresponding boxplots are shown.

Table 8. Overall performance of bivariable models. The Signature Skill Score (SSS) was computed using 100 random signatures that were generated additionally and did not contain Random and NPI+Random signatures.

	C-index	IAUC	SSS	Nagelkerke's R^2
**Training set (METABRIC)**				
**NPI + Developed Signature Score**	0.743	0.773	0.132	0.161
**NPI + Random Signature Score**	0.691	0.697	0.049	0.095
**Test set 1 (METABRIC)**				
**NPI + Developed Signature Score**	0.696	0.693	0.001	-
**NPI + Random Signature Score**	0.676	0.675	0.060	-
**Test set 2 (GSE96058)**				
**NPI + Developed Signature Score**	0.629	0.634	0.083	-
**NPI + Random Signature Score**	0.601	0.568	0.047	-

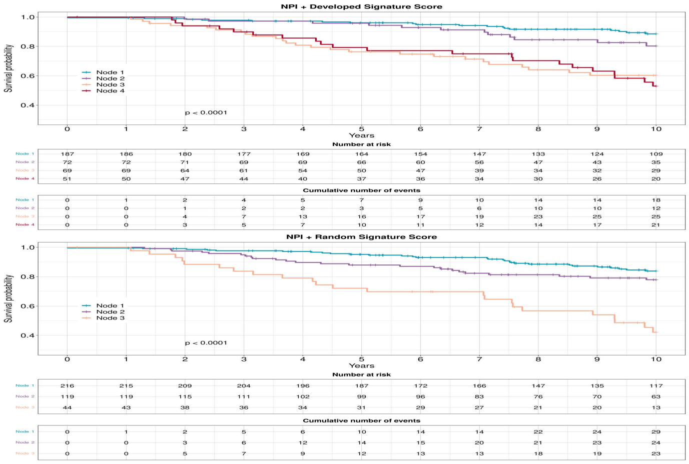

Figure 13. Survival curves of no chemo patients in the test set 1 (METABRIC) with respect to risk classifications for the NPI+Developed Signature Score and NPI + Random Signature Score. Risk groups were identified by Decision Trees. For each signature, the algorithm found different numbers of risk groups indicated by Node 1, Node 2, etc. P-values were calculated from the two-sided logrank test.

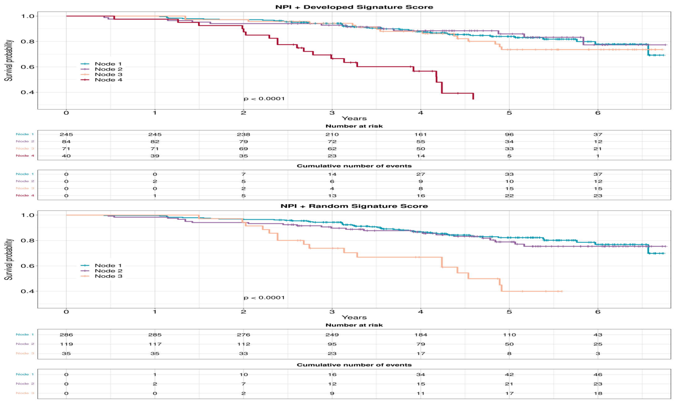

Figure 14. Survival curves of no chemo patients in the test set 2 (GSE96058) with respect to risk classifications for the NPI+Developed Signature Score and NPI + Random Signature Score. Risk groups were identified by Decision Trees. For each signature, the algorithm found different numbers of risk groups indicated by Node 1, Node 2, etc. P-values were calculated from the two-sided logrank test.
