# Supplementary material for: Comparative analysis of molecular signatures reveals a hybrid approach in breast cancer: Combining the Nottingham Prognostic Index with gene expressions into a hybrid signature
Source: PLoS One. 2022 Feb 10;17(2):e0261035. doi: 10.1371/journal.pone.0261035 (PMC8830616; doi:10.1371/journal.pone.0261035)
Supplement: S1 Table — (PDF) [file pone.0261035.s005.pdf]

S1 Table. Descriptive statistics of selected METABRIC data. All variable names listed in the table are taken unchanged from the METABRIC dataset. P-values were computed using the anova test for continuous variables, and the chi-square test for categorical variables.

	Test set 1 (N=379)	Training (N=883)	P-value
Age.at.Diagnosis			0.726
- Mean (SD)	64.467 (11.540)	64.213 (11.880)	
- Range	28.040 - 89.430	26.360 - 92.140	
Cancer.Type.Detailed			0.435
- Breast	3 (0.8%)	9 (1.0%)	
- Breast Angiosarcoma	0 (0.0%)	1 (0.1%)	
- Breast Invasive Ductal Carcinoma	288 (76.0%)	630 (71.3%)	
- Breast Invasive Lobular Carcinoma	26 (6.9%)	87 (9.9%)	
- Breast Invasive Mixed Mucinous Carcinoma	6 (1.6%)	13 (1.5%)	
- Breast Mixed Ductal and Lobular Carcinoma	53 (14.0%)	127 (14.4%)	
- Invasive Breast Carcinoma	3 (0.8%)	16 (1.8%)	
ER.Status			
- Positive	379 (100.0%)	883 (100.0%)	
PR.Status			0.843
- Negative	115 (30.3%)	263 (29.8%)	
- Positive	264 (69.7%)	620 (70.2%)	
HER2.Status			
- Negative	379 (100.0%)	883 (100.0%)	
Hormone.Therapy			0.700
- NO	117 (30.9%)	263 (29.8%)	
- YES	262 (69.1%)	620 (70.2%)	
Neoplasm.Histologic.Grade			0.021
- N-Miss	17	47	
- 1	61 (16.9%)	93 (11.1%)	
- 2	185 (51.1%)	443 (53.0%)	
- 3	116 (32.0%)	300 (35.9%)	
Nottingham.prognostic.index			0.063
- Mean (SD)	3.610 (1.044)	3.735 (1.107)	
- Range	1.000 - 6.150	1.000 - 6.300	
Overall.Survival..Months.			0.622
- Mean (SD)	133.438 (71.839)	135.710 (76.382)	
- Range	0.100 - 355.200	1.233 - 351.000	
Overall.Survival.Status			0.964
- 0:LIVING	154 (40.6%)	360 (40.8%)	
- 1:DECEASED	225 (59.4%)	523 (59.2%)	
Vital.Status			0.745
- N-Miss	0	1	
- Died of Disease	109 (28.8%)	237 (26.9%)	
- Died of Other Causes	116 (30.6%)	285 (32.3%)	
- Living	154 (40.6%)	360 (40.8%)	
Radio.Therapy			0.214
- NO	165 (43.5%)	418 (47.3%)	
- YES	214 (56.5%)	465 (52.7%)	
Chemotherapy			
- NO	379 (100.0%)	883 (100.0%)	
Tumor.Size			0.388
- N-Miss	4	9	
- Mean (SD)	24.294 (11.656)	24.967 (13.028)	
- Range	1.000 - 99.000	1.000 - 150.000	
