# Supplementary material for: Comparative analysis of molecular signatures reveals a hybrid approach in breast cancer: Combining the Nottingham Prognostic Index with gene expressions into a hybrid signature
Source: PLoS One. 2022 Feb 10;17(2):e0261035. doi: 10.1371/journal.pone.0261035 (PMC8830616; doi:10.1371/journal.pone.0261035)
Supplement: S2 Table — (PDF) [file pone.0261035.s006.pdf]

S2 Table. Descriptive statistics of selected GSE data. All variable names listed in the table are taken unchanged from the GSE96058 dataset. P-values were computed using the anova test for continuous variables, and the chi-square test for categorical variables.

	Downsampled test set (N=440)	Full test set (N=1381)	P-value
age at diagnosis:ch1			0.168
- Mean (SD)	64.734 (9.499)	64.015 (9.533)	
- Range	37.000 - 79.000	34.000 - 79.000	
chemo treated:ch1			
- 0	440 (100.0%)	1381 (100.0%)	
endocrine treated:ch1			0.948
- 0	60 (13.6%)	190 (13.8%)	
- 1	380 (86.4%)	1191 (86.2%)	
er status:ch1			
- 1	440 (100.0%)	1381 (100.0%)	
her2 status:ch1			
- 0	440 (100.0%)	1381 (100.0%)	
lymph node group:ch1			0.101
- 1to3	82 (18.6%)	291 (21.1%)	
- 4toX	16 (3.6%)	25 (1.8%)	
- NodeNegative	329 (74.8%)	1031 (74.7%)	
- SubMicroMet	13 (3.0%)	34 (2.5%)	
lymph node status:ch1			0.791
- NodeNegative	342 (77.7%)	1065 (77.1%)	
- NodePositive	98 (22.3%)	316 (22.9%)	
overall survival days:ch1			0.013
- Mean (SD)	1610.605 (505.257)	1676.004 (470.396)	
- Range	156.000 - 2468.000	156.000 - 2473.000	
overall survival event:ch1			< 0.001
- 0	352 (80.0%)	1293 (93.6%)	
- 1	88 (20.0%)	88 (6.4%)	
pgr status:ch1			0.960
- 0	19 (4.3%)	56 (4.1%)	
- 1	403 (91.6%)	1266 (91.7%)	
- NA	18 (4.1%)	59 (4.3%)	
